# A juvenile case of bow hunter’s syndrome due to a bone fragment from an osteophyte of the atlas

**DOI:** 10.1007/s13760-023-02201-5

**Published:** 2023-01-31

**Authors:** Shuaihao Huang, Qifei Duan, Changxiang Liang, Dong Yin

**Affiliations:** 1 Department of Spine, Guangdong Provincial People’s Hospital (Guangdong Academy of Medical Sciences), Southern Medical University, No.106, Zhongshan 2nd Road, Guangzhou, 510080 Guangdong China; 2 The Second School of Clinical Medicine, Southern Medical University, Guangzhou, 510510 Guangdong China

## Introduction

Bowhunter’s syndrome (BHS) is an uncommon condition in which the vertebral artery (VA) is symptomatically occluded during rotation of the head within the normal physiologic range [1, 2]. It may occur anywhere along with the VA, but C1-C2 levels are particularly vulnerable to compression. Numerous etiologies have been reported for VA compression, but osteophyte is the most common cause, and the left VA is more commonly involved in patients with BHS. As a rare disease, the diagnosis and management of BHS can be difficult at times, and there are only a limited number of reports of BHS, especially in adolescents [3]. Therefore, many doctors in face of such patients are easy to misdiagnose or even miss diagnoses.

In this report, we recorded that when a teenager turned his head to the left, the osteophyte on the left side of the atlas compressed the left VA, followed by a series of related symptoms, and how to manage this process.

## Case report

### History, physical examination, and diagnostic workup

**Fig. 1:**
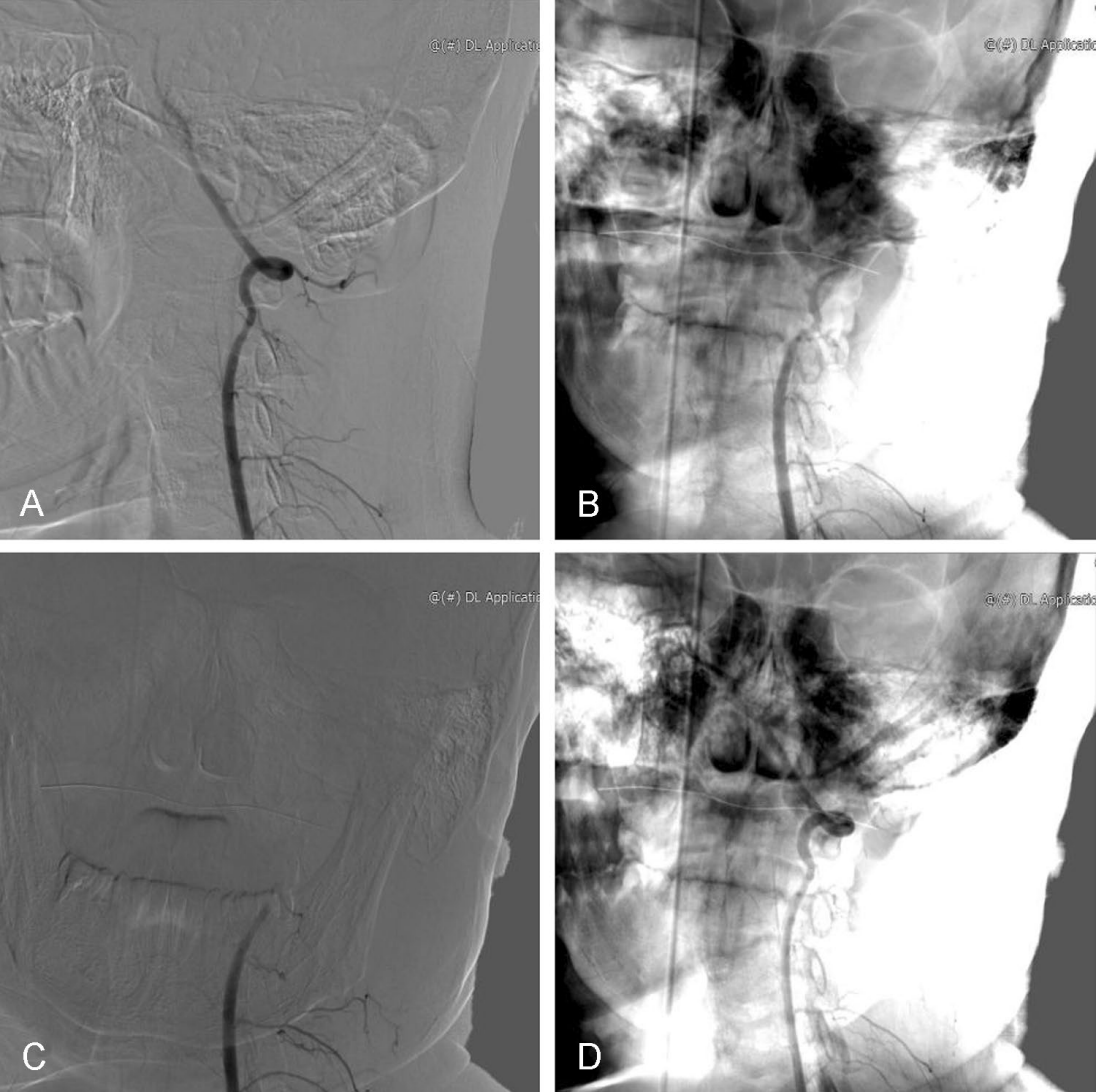
Dynamic digital subtraction angiography of the left vertebral artery. **A** The left vertebral artery is fully patent in the neutral position. **B** The left vertebral artery is narrowed by 40% when the head is rotated 45° to the left. **C** The left vertebral artery occlusion when the head is completely rotated to the left. **D** The left vertebral artery is fully patent again when the head is held back in the neutral position

**Fig. 2:**
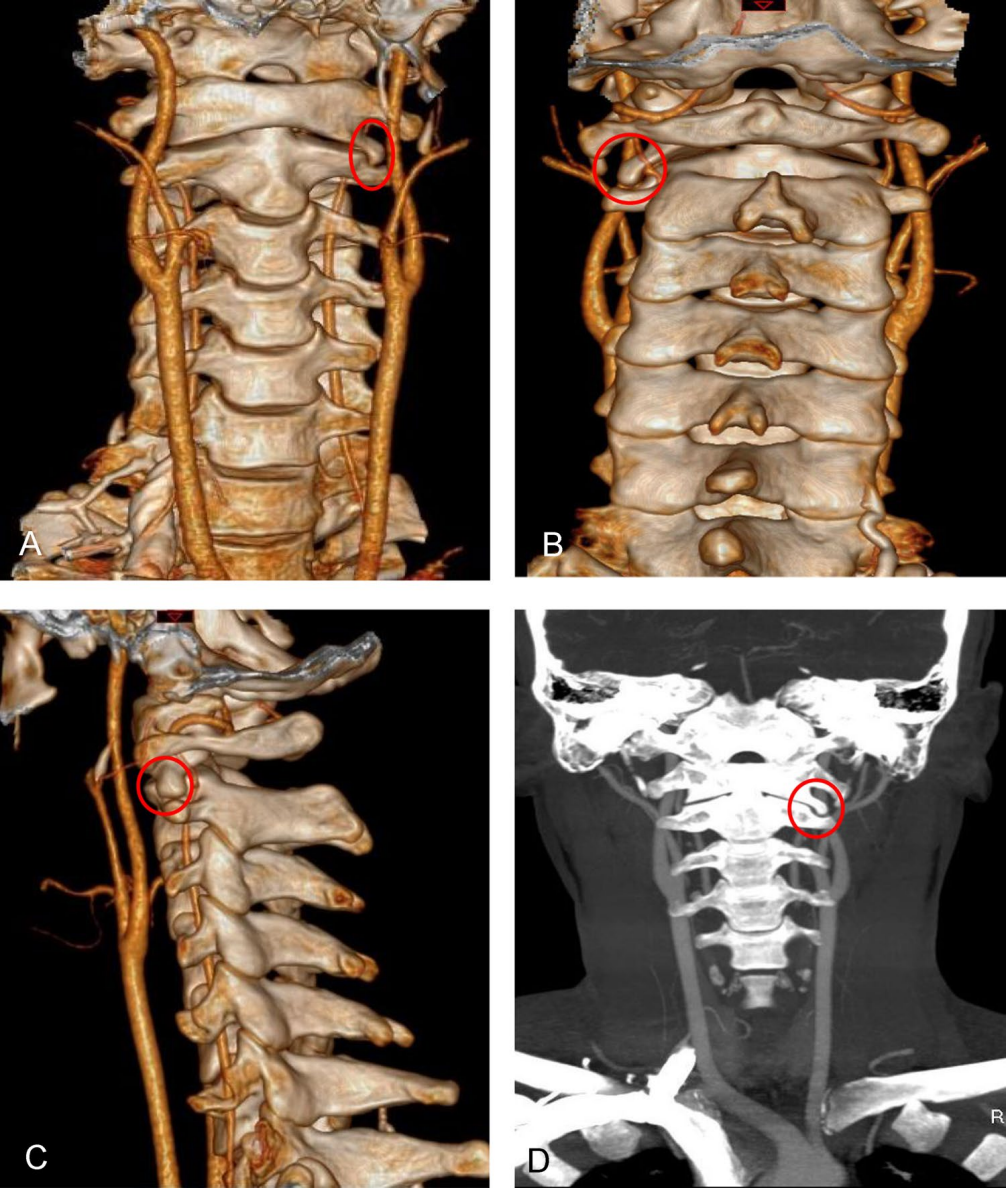
Preoperative 3D-computed tomography and Computed tomography angiography. Preoperative 3D-computed tomography (**A**, **B**, **C**) of the patient’s neck showing the precise anatomical location of the C1 left osteophyte (red circle). Preoperative Computed tomography angiography (**D**) of the patient’s neck showed no sign of bilateral vertebral artery stenosis in the neutral position, and the C1 left osteophyte (red circle) could be seen (Color figure online)

The patient, an 18-year-old teenager, complained of syncope, vertigo, disequilibrium, and consciousness. He first noticed these symptoms two years ago when rotating his head to the left while running outside. For two years, he’s had these symptoms every time he turned his head to the left, which would resolve almost immediately on the return of his head to the neutral position. These symptoms became more frequent recently, so he sought medical attention. He had no special past medical history, social history, and family history, as well as no relevant past surgical history. His neurological examination was normal and there were no neurological defects. The patient was first referred to neurosurgery, MRI revealed subacute infarction of the left cerebellum. After the cardiovascular disease was ruled out in the department of cardiology, secondary prophylactic drugs for the cerebrovascular disease were given. Computed tomography angiography (CTA) and magnetic resonance angiography (MRA) were performed when symptoms recurred. The results showed that there was no sign of bilateral vertebral artery stenosis in the neutral position. He underwent a dynamic digital subtraction angiography (dDSA) to further elucidate an underlying etiology and the results showed that there was no stenosis of the left VA in the neutral position, but progressed to full occlusion at the V2 segment of VA with left head rotation (Fig. 1). According to the results of dDSA, the above symptoms were considered to be caused by dynamic compression of the patient’s VA. Therefore, the patient was referred to spine surgery to determine whether the cause was of cervical origin. 3D-computed tomography (3D-CT) of the cervical spine had been done and showed compression of the vertebral artery by the left osteophyte of the atlas (Fig. 2).

### Surgery and postoperative follow-up

Based on the patient’s symptoms, course of the disease, and imaging findings, after discussing different treatment options with the patient, we chose the surgical treatment. A part of C2 transverse foramen unroofing and C1 osteophyte resection without fusion via a posterior approach was performed. Under a microscope, the left C1/2 facet border was exposed and the C1 osteophyte was noted to severely compress the left VA. Using piezosurgery, the upper dorsal portion of the C2 transverse foramen was resected to release the VA and create operating space for C1 osteophyte resection. A large osteophyte was removed and the VA pulse was bounding. None of the C1/2 facets was removed, which did not influence the stability, therefore, fusion was no need to be performed.

**Fig. 3:**
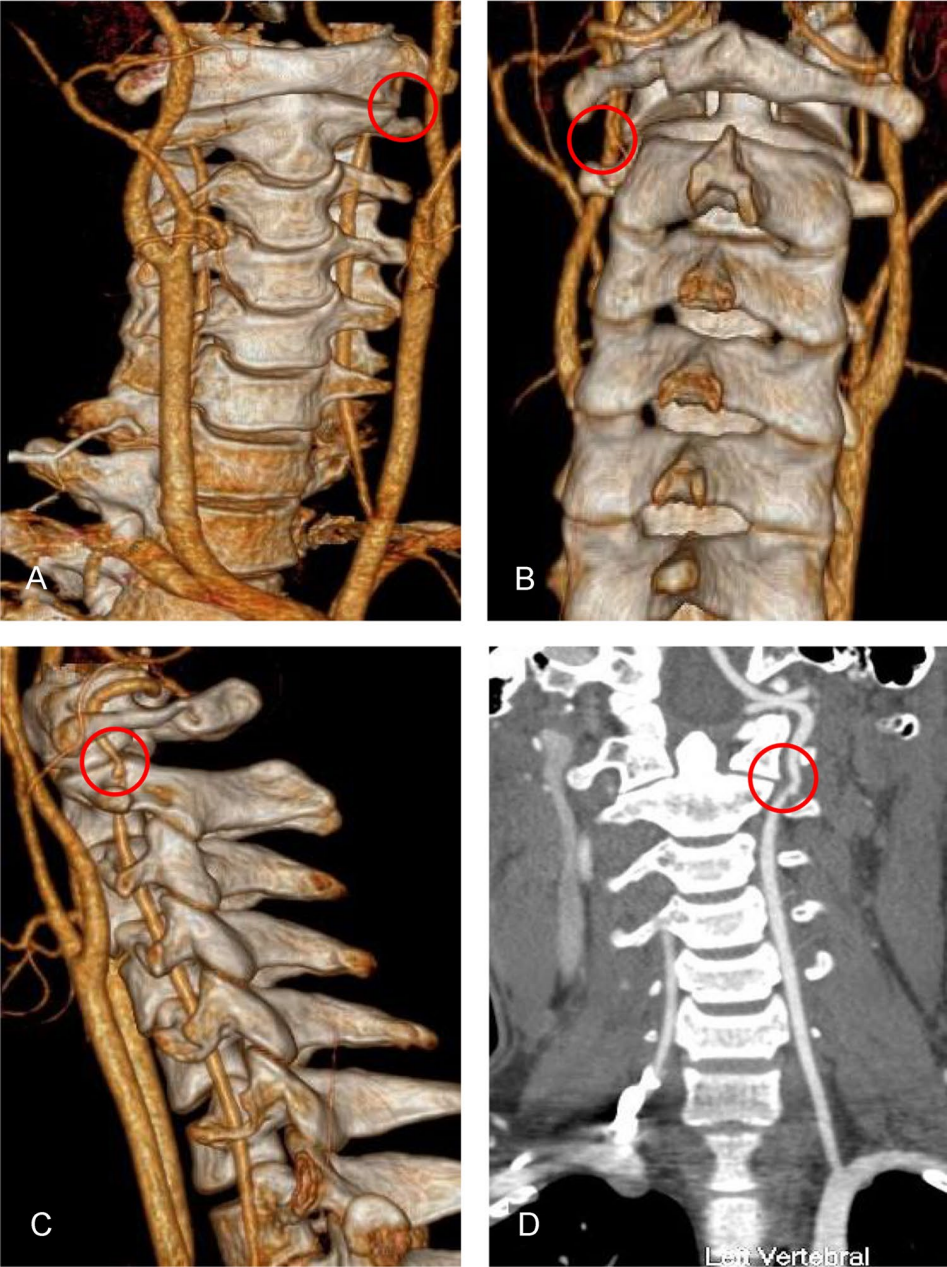
Postoperative 3D-computed tomography and Computed tomography angiography. Postoperative 3D-computed tomography (**A**, **B**, **C**) of the patient’s neck confirmed resection of the osteophyte and a part of C2 transverse foramen unroofing for the left vertebral artery decompression (red circle). Postoperative Computed tomography angiography (**D**) of the patient’s neck showed the left vertebral artery was fully decompressed and the C1 left osteophyte had been removed (red circle) (Color figure online)

The patient tolerated surgery well and noted complete resolution of his preoperative symptoms after surgery. Postoperative 3D-CT confirmed resection of the osteophyte and a part of C2 transverse foramen unroofing for the left VA decompression (Fig. 3). He recovered well and was discharged on postoperative day 5. He remained asymptomatic at 2 months of follow-up.

## Discussion

Bowhunter’s syndrome which was named in 1978 is a rare neurovascular condition characterized by insufficiency of the posterior cerebral circulation induced by rotation of the head within the normal physiologic range [4]. Causes of BHS include osteophyte, diffuse idiopathic skeletal hyperostosis (DISH), cervical spondylosis, disc herniation, fibrous bands, soft tissue hyperplasia, an abnormal VA, or tumors, but osteophyte is the most common cause of vertebral artery occlusion, and left vertebral artery is more commonly involved in patients with Bow Hunter's syndrome [3]. Multiple imaging modalities have been used for the diagnosis of BHS which include transcranial Doppler sonography (TCD), CTA, MRA, and dDSA, however, dDSA has been widely regarded as the gold standard [3].

Treatment options in BHS depend on its etiology, the severity of the case, and patient circumstances. Conservative treatment includes cervical collars and avoiding prolonged head rotation as well as antiplatelet or anticoagulation therapies. Surgical options include cervical decompression or fusion, and endovascular stents to increase blood flow during head-turning [3]. Studies by Rastogi et al. have found that surgery is more effective than other treatment modalities [3]. The most common cause of BHS is compression of the bone structure, which can be quickly relieved by surgery, thus relieving symptoms. Therefore, surgery should be the primary treatment for BHS patients, especially those with bone compression as the cause.

In regards to our patient, 3D-CT showed C1 left osteophyte compressed the VA, and dDSA showed nearly 100% stenosis at the V2 segment of the left VA with left head rotation. After conservative treatment failed, we surgically removed the osteophyte that was compressing the VA to relieve rotational compression and achieved satisfactory short-term follow-up. We believe that dDSA is the gold standard for diagnosing BSH. For these patients, especially those with bone compression as the cause, surgery should be the main treatment.
